# Effects of a 12-Week Physical Activity Intervention on Psychological Symptoms in Adolescents

**DOI:** 10.3390/ijerph21121558

**Published:** 2024-11-25

**Authors:** Maria Carolina Juvêncio Francisquini, Géssika Castilho dos Santos, Thais Maria de Souza Silva, Pedro Henrique Garcia Dias, Claudinei Ferreira dos Santos, Gabriel Pinzon, Aristides M. Machado-Rodrigues, Antonio Stabelini Neto

**Affiliations:** 1 Health Sciences Center, State University of Northern Paraná, Jacarezinho 86400-000, Brazil; gessika.castilho@gmail.com (G.C.d.S.); thais.msouza@outlook.com (T.M.d.S.S.); ph.garciadias@gmail.com (P.H.G.D.); neief@uenp.edu.br (C.F.d.S.); pinzonjiu@gmail.com (G.P.); 2 Faculty of Sport Sciences and Physical Education, University of Coimbra, 3040-248 Coimbra, Portugal; rodriguesari@hotmail.com

**Keywords:** mental health, exercise, adolescence, controlled clinical trial, students

## Abstract

Participation in regular physical activity (PA) is associated with numerous health benefits, including improvement in adolescents’ mental health. The current study aimed to assess the effects of a 12-week physical activity intervention on psychological symptoms of adolescents. The sample of this controlled randomized study was composed by 150 adolescents aged 12 to 15 years old. PA intervention is an adaptation of the ActTeens Program, which includes structured PA sessions delivered during Physical Education classes. The *Strengths and Difficulties Questionnaire* (SDQ) was used to measure psychological symptoms. To analyze the effects of the intervention on psychological symptoms, generalized estimating equations (GEEs) models were constructed. After 12 weeks, there were no significant intervention effects for emotional problems (mean difference: −0.14; 95% IC: −1.1–0.82), conduct problems (mean difference: 0.14; 95% IC: −0.6–0.8), attention deficit/hyperactivity disorder (mean difference: −0.66; 95% IC: −1.4–0.1), peer problems (mean difference: 0.2; 95% IC: −0.6–1.0), prosocial behavior (mean difference: 0.00; 95% IC: −0.8; 0.8) and overall mental health score (mean difference: 0.15; 95% IC: −2.0; 2.3). No improvement in psychological symptoms was observed after 12 weeks of PA intervention. Future studies should explore how PA in different dimensions and contexts may impact positively on adolescents’ mental health.

## 1. Introduction

Adolescence is characterized as a period of greater vulnerability for mental disorders [1], since significant biological, social, and psychological changes occur and many social interactions are impacted [2]. It is estimated that 12–14% of the world’s adolescents live with a mental disorder, in which depression (1.8%), anxiety (5.2%), attention deficit hyperactivity disorder (3.7%) (ADHD) and conduct problems (1.3%) are the most prevalent among this population [1,3,4]. In the Brazilian population, the population-based study (ERICA) demonstrated that approximately 30% of adolescents aged 12 to 17 years have some type of mental health disorder [5].

According to the World Health Organization [6], mental disorders could become the leading public health issue by 2030. In fact, mental disorders may have adverse implications for an individual’s social and educational competencies [1]. In addition, these conditions can increase the risk of developing subsequent psychiatric outcomes and suicidal ideation [7]. This emphasizes the need for further investigations about how to enhance adolescents´ mental health. Previous research has demonstrated significant effects of physical activity (PA) on adolescents’ mental health, where higher PA frequency (6 to 7 days per week) was significantly associated with improvements in overall mental health [8], reduction in depression, anxiety [9] and hyperactive/inattentive symptoms [8], and increase in self-concept and self-esteem [10].

PA plays a physiological role, since it may complement some medications if correctly prescribed. The literature has revealed that PA engagement can increase levels of norepinephrine, dopamine, and serotonin [11], since imbalances in these neurotransmitters are precursors of psychological symptoms such as anxiety [12], depression [13] and ADHA [14]. Furthermore, other mechanisms may contribute to the improvement in psychological outcomes, such as psychosocial, in which it is postulated that PA provides an opportunity for social interaction, mastery in the physical domain, improvements in appearance self-perceptions, and independence [15]. For example, previous studies showed that PA can promote social interaction, which can reduce symptoms related to conduct problems, antisocial behavior, perceived appearance and physical self-concept [13,14,16], as well as a decreased risk of negative thoughts and psychological distress [17]. Other mechanisms are the behavioral mechanisms, in which it is proposed that changes in mental health outcomes resulting from PA are mediated by changes in relevant and associated behaviors, such as sleep time and PA [8,15], since sleep is causally related to the experience of mental health difficulties, and insufficient sleep was associated with deficits in cognitive function and an increase in behavioral problems [15,18].

Regarding mental health outcomes and PA interventions, the results are unclear. Leahy et al. [19] conducted a 14-week intervention, and found that intervention participants demonstrated improvements in specific subscales of emotional problems and peer problems in comparison with the control group. On the other hand, Wassenaar et al. [20] conducted a high-intensity intervention among school-aged adolescents over the course of one year, and no significant changes were found in cognitive performance or mental health outcomes (internalizing, externalizing symptoms) in adolescents. Eather et al. [16] also found no significant differences in these psychological difficulties after an 8-week resistance training program.

Lubans et al. [15] postulated that components of physical activity, such as time and frequency, may moderate the physical activity-induced benefits for mental health in children and adolescents. Intensity and intervention sessions (short- term vs. long-term) can serve as moderators between physical activity interventions and mental interventions [21]. A recent meta-analysis conducted by Liu et al. [21] found that physical activity interventions with more sessions in total, longer total duration, or higher frequency generated greater benefits for overall mental health. For example, studies by Leahy et al. [19] and Ganjeh et al. [8] demonstrated improvements in specific subscales of emotional problems, peer problems and hyperactive symptoms post intervention, lasting 12 or more weeks. Bell et al. [22] corroborates these findings, noting a protective effect of PA in the emotional problems subscale. Liu et al. [21] also found that aerobic exercises provided greater benefits for internalizing problems, since the aerobic exercise process facilitates the release of endorphins and the increase in neurotransmitters, which may explain the greater benefits of aerobic exercise for psychological benefit [23].

Interventions in the school environment have great potential to increase adolescents’ participation in PA, considering that young people spend a significant portion of their day in the school setting, where they acquire knowledge on various topics, including health [24,25]. Furthermore, physical education classes, in addition to developing physical practices and motor skills, also contribute to improvements in physical and mental well-being [26]. A recent systematic review and meta-analysis [24] showed that school-based PA interventions are promising strategies for improving mental health outcomes in adolescents. However, Wassenaar et al. [20] found no significant improvements in psychological difficulties after a high-intensity exercise program incorporated into regular physical education (PE) classes. Despite some benefits of PA for psychological symptoms in adolescents, there are still few intervention studies focused on these outcomes among Latin American adolescents and those from developing countries, such as Brazil. This gap is evidenced by a lack of basic conditions, such as trained staff, quality materials, and suitable environments for PA practice [27]. Therefore, the aim of the present study was to assess the effects of a 12-week school-based PA intervention on self-reported adverse psychological symptoms in school-aged adolescents. We hypothesized that the intervention group would show improved psychological symptoms compared to the control group.

## 2. Materials and Methods

### 2.1. Participants and Procedure

This study adopted a 12-week randomized controlled trial design, following Consolidated Standards of Reporting Trials (CONSORT) recommendations [28]. The trial was approved by the human research ethics committee of the State University of Northern Parana, Brazil (Registration No. 4.452.513) and registered in Clinical Trials (NCT05070377).

**Figure 1:**
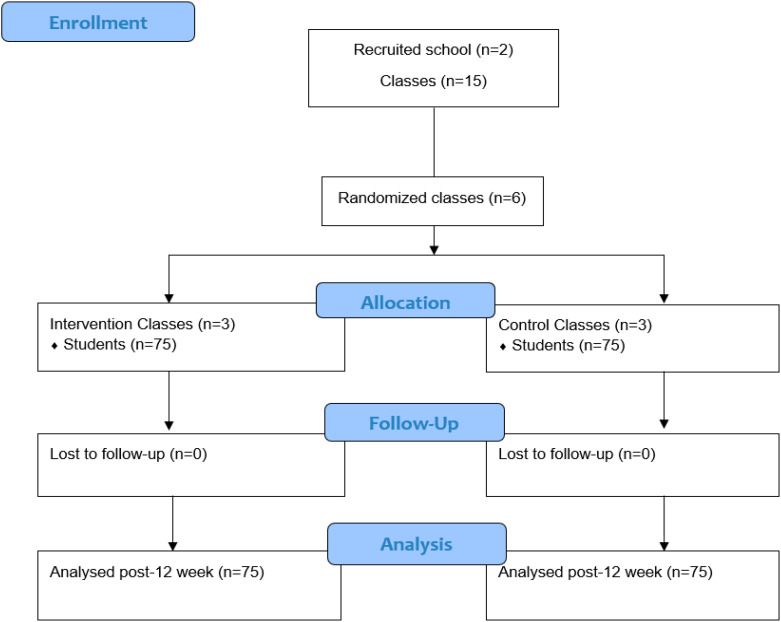
Analyzed post 12 weeks.

Secondary public schools from Jacarezinho City, Brazil, including students aged between 12 and 15 years old (i.e., Grades 8 and 9) were eligible to participate in the study. The schools were recruited through a list provided by the Regional Education Center. Then, emails were sent directly to the eligible schools. Four schools were invited to participate in the study; however, only two expressed interests. Inclusion criteria for the schools were being secondary level, having at least one class of the 8th and 9th grades, and Physical Education (PE) classes two times a week. A member of the research team met with the agent of each school who was interested in taking part to explain the study. The criterion for participant exclusion was not taking part in all stages of the study. The adolescents and their parents/guardians gave written consent to participate in this research. For this trial, sample size calculation was based on effect size of 0.10; power of 80%; confidence level of 5%: and correlation coefficient as 0.05. Considering an attrition of 10%, a minimum of 150 students was required. Considering an average of 25 students per class, six classes were randomized by an independent researcher to either a control or an intervention condition using a computer-based random-number generator (see Figure 1—flow diagram).

### 2.2. Intervention

The intervention is an adaptation of the multicomponent school-based PA program ActTeens Program [29], which included structured physical exercise sessions in the school environment. The structured sessions were implemented over 12 weeks (August–October 2023) and delivered within PE lessons, twice a week, for 20 min per lesson. The sessions aimed to promote opportunities to practice non-traditional PA in the school context, encouraging the adoption of daily active behavior, and reinforcing the importance of an active lifestyle throughout adolescence. The sessions consisted of a combination of muscle-strengthening and aerobic exercise.

The session followed a specific format, including warm-up with movement-based games (3 min); physical exercise (15 min); and cool-down, including static stretching (2 min). In each session, participants were free to choose their own groups (groups of 4–5 people) and were free to choose the sequence of exercises they would like to perform (4–5 exercises), from a variety of cards, incorporating aerobic (i.e., running on the spot, jumping rope and lateral movement) and resistance exercises (i.e., squats, bent-over row and biceps curl). The intensity of the exercises was monitored via heart rate (HR) sensors (Polar Verity Sense), which were connected to a central iPad application (Polar Team). The participants wore the equipment on their right upper arm. The HR monitors were numbered so that each student used the same equipment in every session, and they were removed right after the end of the session. During the physical exercise, the students were verbally encouraged by the leading researcher to reach an intensity equal to or greater than 70% of their estimated maximum HR. The session design has been previously described elsewhere [29].

### 2.3. Measures and Data Collection

All assessments were conducted at the school by trained research assistants, who were blinded to group allocation at all time points (baseline and post-intervention). Data were collected in the second half of 2023 (baseline: July 2023; post-intervention: November 2023) during PE classes. Self-report information was assessed using specific questionnaires. Two researchers of both sexes conducted anthropometric assessments. The data collection proceeded as follows: (1) questionnaires regarding personal information (sex, age, parental education level), physical activity and psychological symptoms; (2) anthropometric assessments (i.e., body weight, height); and (3) cardiorespiratory fitness (CRF) assessments.

#### 2.3.1. Mental Health

Psychological symptoms were assessed using the Strengths and Difficulties Questionnaire (SDQ) used for the Brazilian population according to Fleitlich and Goodman [30,31]. The SDQ is a self-report questionnaire consisting of 25 items, divided into 5 subscales: emotional problems, conduct problems, hyperactivity/inattention, peer problems, and prosocial behavior. The reliability of the self-report subscales are 0.82 for the total difficulties, 0.75 for emotional symptoms, 0.72 for conduct problems, 0.69 for hyperactivity, 0.65 for prosocial behavior, and 0.61 for peer problems [32]. The score within these sub-scales ranged from 0 to 10. The total difficulties score was calculated by adding the scores of the sub-scales (emotional problems + conduct problems + hyperactivity/inattention + peer problems), except for the prosocial behavior scale. A higher score indicates a higher difficulty grade.

#### 2.3.2. Physical Activity

PA was assessed using the Physical Activity Questionnaire for Adolescents (PAQ-A), translated to Brazilian Portuguese and validated for Brazilian adolescents [33]. The PAQ-A is a self-administered, 7-day recall questionnaire, which assesses participation in different PAs (e.g., during PE classes, lunch break, after school, in the evenings and at weekends). The questionnaire consists of eight questions structured to discern low (score 1) to high (score 5) PA. The total score of this questionnaire was calculated by adding all questions’ average scores. The PAQ-A presents internal consistency of α = 0.76, and intraclass correlation coefficient of 0.78 [33].

#### 2.3.3. Cardiorespiratory Fitness

CRF was assessed using the PACER test [34], which is valid and reliable for this population [35,36]. A 20 m course was set up indoors on a hard surface with students instructed to run back and forth between two lines following an accompanying audio file. Test administrators provided verbal encouragement to participants to maximize their motivation. The test was ended when the participant failed to complete two consecutive laps in the allotted time or voluntarily dropped out due to fatigue. The total number of laps was registered and used as cardiorespiratory fitness outcome.

### 2.4. Data Analysis

The general characteristics of the participants were presented as means (standard deviations) for continuous variables, and proportions for the categorical data. T-tests were used to identify the possible differences between participants within the intervention and control groups at baseline. Generalized estimating equations (GEEs) models were constructed to analyze the effect of the intervention on the changes in mental health (intra-group). These models are considered appropriate for continuous response variables and repeated measures, reflecting the association between the response and the independent variables. The GEE model was constructed for each indicator, and the corrected quasi-likelihood under the independence model criterion (QICc) was used to evaluate the fit of the model to the data. The lower the QICc, the better the fit of the model. The analyses were adjusted for the following variables: sex, age, PA and CRF at baseline. The data analyses were performed using the statistical software package *Statistical Package for the Social Sciences* Version 25 (IBM, New York, NY, USA), with significance set at *p* < 0.05.

## 3. Results

The study sample comprised 150 adolescents (52.9% females). Table 1 provides the participants’ characteristics. No significant differences were observed between the groups at baseline.

After 12 weeks, adolescents of the IG revealed no significant reductions in emotional problem symptoms (mean diff: −0.36; IC 95%: −1.05; 0.33), conduct problems (mean diff: 0.41; IC 95%: −0.17; 0.99), ADHD (mean diff: −0.07; IC 95%: −0.61; 0.47), peer problems (mean diff: 0.31; IC 95%: −0.29; 0.91), prosocial behavior (mean diff: −0.07; IC 95%: −0.74) and total difficulties score (mean diff: 0.57; IC 95%: −0.86; 2.01) (Table 2). For the control group, we did not find significant changes in emotional problems (mean diff: −0.21; IC 95%: −0.88; 0.46), conduct problems (mean diff: 0.27; IC 95%: −0.20; 0.75), ADHD (mean diff: 0.59; IC 95%: 0.02; 1.16), peer problems (mean diff: 0.11; IC 95%: −0.50; 0.72), prosocial behavior (mean diff: −0.07; IC 95%: −0.59; 0.44) and total difficulties score (mean diff: 0.41; IC 95%: −1.16; 1.99).

Furthermore, when the intervention effects between two groups were verified (Table 2), no significant effect was found for emotional problem symptoms (mean diff: −0.14; IC 95%: −1.1; 0.82; p: 0.76), conduct problems (mean diff: 0.14; IC 95%: −0.6; 0.8; *p*: 0.71), ADHD (mean diff: −0.66; IC 95%: −1.4; 0.1; *p*: 0.10), peer problems (mean diff: 0.2; IC 95%: −0.6; 1.0; *p*: 0.64), prosocial behavior (mean diff: 0.00; IC 95%: −0.8; 0.8; *p*: 0.98) and total difficulties score (mean diff: 0.15; IC 95%: −2.0; 2.3; *p*: 0.88).

## 4. Discussion

The aim of the study was to assess the effects of a PA intervention on psychological symptoms in adolescents. There were no significant changes in the emotional problems, conduct problems, ADHD, peer problems, prosocial behavior, and total difficulties score post intervention. The literature supports the fact that PA engagement can lead to improvements in psychological outcomes of adolescents [11]; PA induces an increase in central excitation, which is commonly associated with the release of dopamine, norepinephrine, and serotonin [8,22], and these hormones are primarily involved in the regulation of emotional functions, reward-related processes and cognitive function [37]. Moreover, PA engagement provides opportunities for social interaction, and improvements in social relationships can influence the decrease of internalizing and externalizing disorder symptoms [38].

However, considering the duration of the intervention/session, studies showed that longer PA interventions [21,39], with higher frequency (three times/week) lasting 30–40 min have been found effective in improving mental health outcomes, including cognitive function, psychological well-being, and internalizing and externalizing problems [21,39]. However, it is important to highlight the fact that although our study has a long duration, it included only 20 min sessions two times per week. Furthermore, research also indicates that sessions involving higher-intensity activities combined with sports elements may be more effective, as this type of activity can provide enjoyment and confidence as individuals progress in performing the exercise [19,40].

Furthermore, considering that adolescents spend most of their day in the school environment, school appears to be the ideal setting for developing physical activity interventions [24]. Previous school-based PA interventions have found improvements in psychological symptoms, such as overall psychological difficulties, emotional problems, and peer problems [24,25]. For example, Eather et al. [16] conducted an intervention with 96 adolescents for 8 weeks. They found that intervention participants categorized as “at risk” of psychological distress demonstrated improvements in self-esteem, perceived appearance, physical self-concept and total difficulties score. Additionally, Lubans et al. [41] conducted a 20-week intervention with adolescents considered “at risk” of obesity, involving activities focused on muscular fitness with 90 min sessions. The results demonstrated that the intervention on psychological well-being was small but statistically significant. However, the present study sample was not categorized as “at risk” of psychological distress. In addition, most participants exhibited values considered “satisfactory” at baseline (Table 1), limiting the potential for further improvements as a result of the intervention. Furthermore, the type and intensity of PA proposed in effective interventions involved high-intensity exercise [19]. For example, Leahy et al. [19] conducted an intervention with HIIT protocol, with three sessions per week (12–20 min) for 14 weeks, and found a reduction in total psychological difficulties score, and in two specific subscales (“emotional problems” and “peer problems”).

PA interventions of same session duration but different intensity may generate different effects. For example, Smith et al. [42] examined the impact of a school-based Resistance Training for Teens intervention on adolescents’; however, no significant results were found for the assessed mental health markers. On the other hand, Leahy et al. [19] using a high-intensity training strategy, found significant results for mental health outcomes. Participation in high-intensity activity can be a less monotonous strategy compared to other PA programs, providing enjoyment and potentially leading to improvements in mental health symptoms among youth [43], whereas the present study utilized moderate-to-vigorous intensity exercise.

Our intervention was designed to address the individual’s basic psychological needs [44], incorporating the following strategies: (1) creating opportunities for engaging in moderate-to-vigorous physical activity in groups (relationship): students had the chance to form their own groups based on their level of affinity with peers; (2) support from teachers and staff (competence): students received correction and encouragement from teachers and staff regarding the practice and execution of exercises; and (3) autonomy: at this stage, students, in groups, had the opportunity to choose their own circuit from a variety of pre-established cards. Despite our belief that these strategies could positively impact mental health outcomes, our hypothesis was refuted. Additionally, the 20 min session used in the current study may be an important aspect, because some studies applied longer sessions (60 min) [16].

Although the current intervention had been designed to satisfy the basic psychological needs of the adolescents (i.e., autonomy, competence and relatedness), and lead to improvements in psychological symptoms [16,19], this type of exercises might not be interesting to this population, limiting the potential benefits for psychological outcomes. According to prior research, participating in team sports may contribute to the development of social skills among adolescents, enhancing their self-esteem and social abilities [11]. Hoffmann et al. [45] found that participation in organized team sports reduces significantly the occurrence of mental difficulties in adolescents. Additionally, Leahy and colleagues [19] showed that participants enrolled in a variety of HIIT workouts (i.e., Sport HIIT, Combat HIIT, and Dance HIIT) reported significant reductions in psychological difficulties after 14 weeks of intervention [19].

The lack of positive results in psychological symptoms among the adolescents of the current study may be related to the fact that our intervention used only one strategy (exercise during PE classes), whereas other intervention studies used strategies that combined PA with extracurricular activities (i.e., outdoor sports activities) [24,46]. Ahmed et al. [46] conducted a 12-week multicomponent intervention that consisted of a health education lesson for 10 min, followed by a 30 min supervised circuit session delivered in the PE, and finally, 20 min of outdoor sports activities of their choice. However, the proposal in the present study only used non-conventional structured PA sessions, without a sports component or extracurricular outdoor activities.

Considering the studies presented, the current study did not find significant improvements after 12 weeks of a PA intervention on psychological symptoms: emotional problems, hyperactivity, conduct problems, peer problems, prosocial behavior, and total difficulties. This may be due to the intensity of the activities, in contrast to studies that reported benefits with high-intensity activities. Future studies should explore multicomponent strategies, such as high-intensity exercises combined with sports elements, opportunities outside the school setting, and increased access to PA for youth experiencing emotional vulnerability.

The study has some strengths. Initially, according to our literature review, no randomized controlled trial was found that explored the use of moderate-to-vigorous PA interventions and their effects on psychological symptoms in Brazilian adolescents. In addition, all sessions were monitored by heart rate sensors to control the intensity of the exercise. Finally, the present study includes the RCT study design. Nevertheless, a limitation should be mentioned. The present study analyzed a sample of two public schools, involving a small sample size, which limits the generalizability of the findings. Another limitation was the use of a self-report questionnaire to measure psychological symptoms, which have well-known disadvantages such as memory bias.

Finally, the results should be interpreted with caution and their implications should be discussed within the broadest possible context. The present study offers directions for future research, such as using strategies that combine structured PA with elements of team sports and outdoor activities. Future research could also focus on adolescents in vulnerable situations (e.g., social and emotional), and with a longer follow-up.

## 5. Conclusions

The current scenario of school-aged youth presenting psychological symptoms urges the implementation of PA interventions in the school environment as a health protective factor, and, considering the aforementioned contrasting results, this should become a research and public health priority. Our findings showed that 20 min of moderate-to-vigorous PA during PE classes did not result in significant changes in psychological symptoms of these adolescents. Therefore, future interventions should target adolescents, particularly those with social and emotional vulnerability, and include a longer follow-up period to advance knowledge about the impact of PA interventions on psychological symptoms in adolescents.

## Figures and Tables

**Table 1:** Sample characteristics.

	Intervention	Control	Total
	N = 75	N = 75	N = 150
Age (Years), Mean ± SD	13.69 ± 0.7	13.58 ± 0.5	13.64 ± 0.6
Female, n (%)	43 (57.3)	38 (48.7)	81 (52.9)
Height (cm), Mean ± SD	160.3 ± 7.9	160.7 ± 7.9	160.5 ± 7.8
Body Weight (kg), Mean ± SD	54.4 ± 13.2	53.2 ± 13.7	53.8 ± 13.5
Pacer, laps, Mean ± SD	20.6 ± 10.6	24.05 ± 16.2	22.4 ± 13.9
PA, score (1–5)	2.9 ± 1.1	2.8 ± 0.9	2.8 ± 1.0
Total difficulties, score (1–40)	15.4 ± 5.5	15.08 ± 6.2	15.2 ± 5.8

SD: standard deviation. PA: physical activity.

**Table 2:** Effects of the intervention on psychological symptoms in adolescents.

Variable	Group	Baseline	12 Weeks	Time, *p*	12 Weeks Adj Diff in Change	Group by Time, *p*
Emotional problems	INT	4.0 (3.5; 4.5)	3.6 (3.1; 4.2)	0.31	−0.14 (−1.1; 0.82)	0.76
	CON	4.5 (3.9; 5.2)	4.3 (3.7; 5.0)	0.53		
Conduct problems	INT	2.9 (2.4; 3.3)	3.3 (2.8; 3.8)	0.16	0.14 (−0.6; 0.8)	0.71
	CON	3.0 (2.6; 3.5)	3.3 (2.9; 3.7)	0.25		
ADHD	INT	4.8 (4.3; 5.4)	4.8 (4.3; 5.2)	0.80	−0.66 (−1.4; 0.1)	0.10
	CON	4.8 (4.1; 5.4)	5.4 (4.9; 5.9)	0.04		
Peer problems	INT	3.3 (2.9; 3.7)	3.6 (3.1; 4.1)	0.31	0.2 (−0.6; 1.0)	0.64
	CON	3.4 (2.9; 3.8)	3.5 (3.0; 4.0)	0.73		
Prosocial behavior	INT	6.6 (5.9; 7.2)	6.5 (5.8; 7.2)	0.84	0.00 (−0.8; 0.8)	0.98
	CON	6.6 (6.0; 7.2)	6.5 (6.0; 7.1)	0.77		
Total difficulties, score	INT	14.2 (12.8; 15.6)	14.8 (13.3; 16.2)	0.43	0.15 (−2.0; 2.3)	0.88
	CON	14.9 (13.2; 16.6)	15.3 (13.6; 17.0)	0.60		

## Data Availability

The data presented in this study are available on request from the corresponding author due to ethical reasons.
